# Effectiveness of Clavicula Pro Humero Reconstruction for Elderly Patients: Report of Two Cases

**DOI:** 10.1155/2016/4140239

**Published:** 2016-10-25

**Authors:** Sho Okimatsu, Hiroto Kamoda, Tsukasa Yonemoto, Shintaro Iwata, Takeshi Ishii

**Affiliations:** ^1^Division of Orthopedic Surgery, Chiba Cancer Center, Chiba 260-8717, Japan; ^2^Department of Orthopedic Surgery, Chiba University, Chiba 260-8677, Japan

## Abstract

Clavicula pro humero (CPH) reconstruction is a method that is used after proximal humeral excision. During CPH reconstruction, the ipsilateral clavicle is rotated downward and connected to the preserved distal humerus by using plates and screws. This method is frequently used for reconstruction surgeries involving young patients and has positive outcomes. In this study, we describe two cases of CPH reconstruction that were performed on elderly individuals after wide resection of the proximal humerus; postoperative results from these surgeries were satisfactory. The average Musculoskeletal Tumor Society (MSTS) functional score after surgery was 68.5%, indicating that CPH reconstruction is suitable for not only younger but also elderly patients, particularly those over the age of 65 years.

## 1. Introduction

The proximal humerus is a common site of both primary and metastatic bone tumors [1, 2]. Several techniques have been established for the reconstruction of the proximal humerus after resection of malignant tumors. However, as no single approach has been deemed superior, the approach used is determined on a case-by-case basis by the surgeon. One such reconstructive technique is clavicula pro humero (CPH), which was originally described for reconstruction of the upper extremity in children with limb deficiencies [3]. In this study, we describe the outcomes of CPH reconstruction in two elderly patients. The postoperative results from these surgeries were comparable to those of other reconstructive options. We conclude that CPH is a suitable reconstructive technique not only for the young, but also for some elderly patients after tumor resection from the proximal humerus.

## 2. Case Presentation

*Case 1*. A 67-year-old man presented with pain and swelling in the right shoulder. He was diagnosed to have metastatic thyroid carcinoma at another facility four years ago and was treated with radioactive iodine.

**Figure 1:**
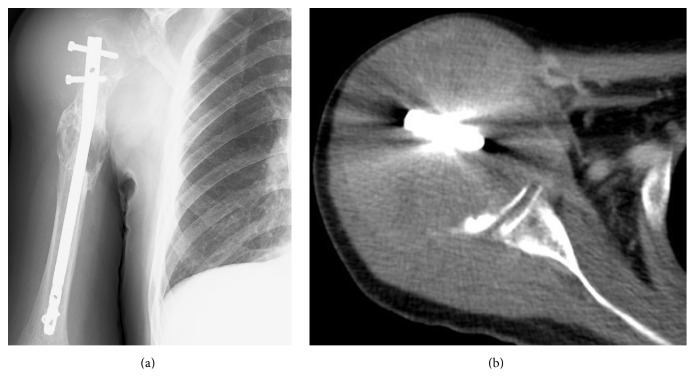
(a) Radiograph and (b) computed tomography (CT) of the right proximal humerus. The lateral cortex of the proximal humerus is absent.

**Figure 2:**
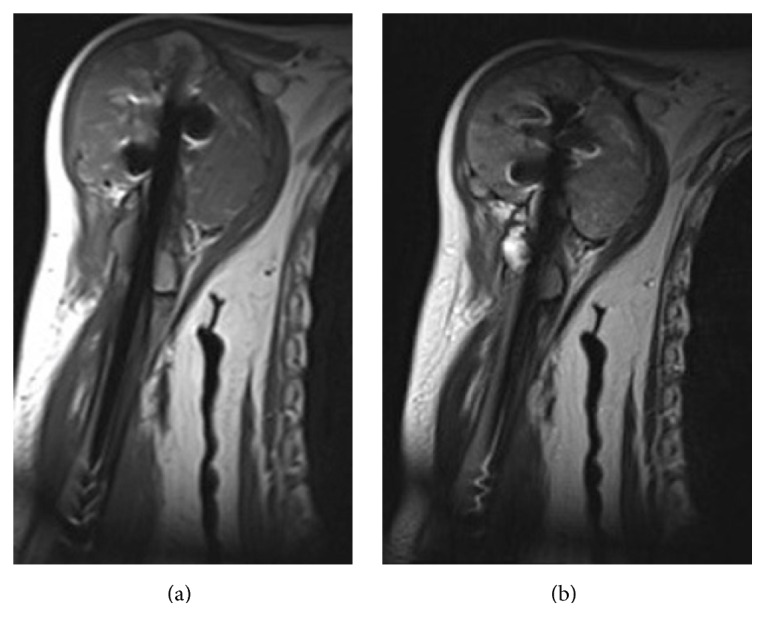
Magnetic resonance imaging (MRI) findings of the right humerus. (a) T1 and (b) T2 weighted imaging show mild high signal changes in the tumor lesion that expanded around the humeral nail.

At initial presentation, he also underwent open reduction and internal fixation of the right proximal humerus for a pathological fracture with the T2 Humeral Nail (Stryker, Kalamazoo, Milwaukee, USA), followed by radiation therapy to the right humerus. He presented to our institution with plain radiographs of his right shoulder showing an osteolytic lesion of the head of the right humerus along with soft tissue swelling of the proximal arm. A computed tomography (CT) scan showed the disappearance of the proximal humeral cortex (Figure 1). Magnetic resonance imaging (MRI) showed the lesion to have mild high signal changes on both T1 and T2 weighted imaging, extending to the surrounding muscles (Figure 2). Needle biopsy confirmed recurrent metastatic thyroid carcinoma. After counseling the patient, a surgical resection and reconstruction were planned. Preoperative embolization of tumor feeding vessels was carried out by interventional radiologists. The surgical incision extended from the medial side of the right clavicle, across the coracoid process, extending to the distal end of the right humerus and included the needle biopsy tract. The tumor and surrounding muscles were carefully resected en bloc with a humeral intramedullary rod. The axillary nerve and posterior humeral circumflex vessels were sacrificed. The right clavicle was mobilized by freeing its medial attachments but leaving its attachments to the acromion intact. The floating clavicle was rotated by 90 degrees clockwise and was fixed to the distal humerus with an AO small fragment locking plate.

**Figure 3:**
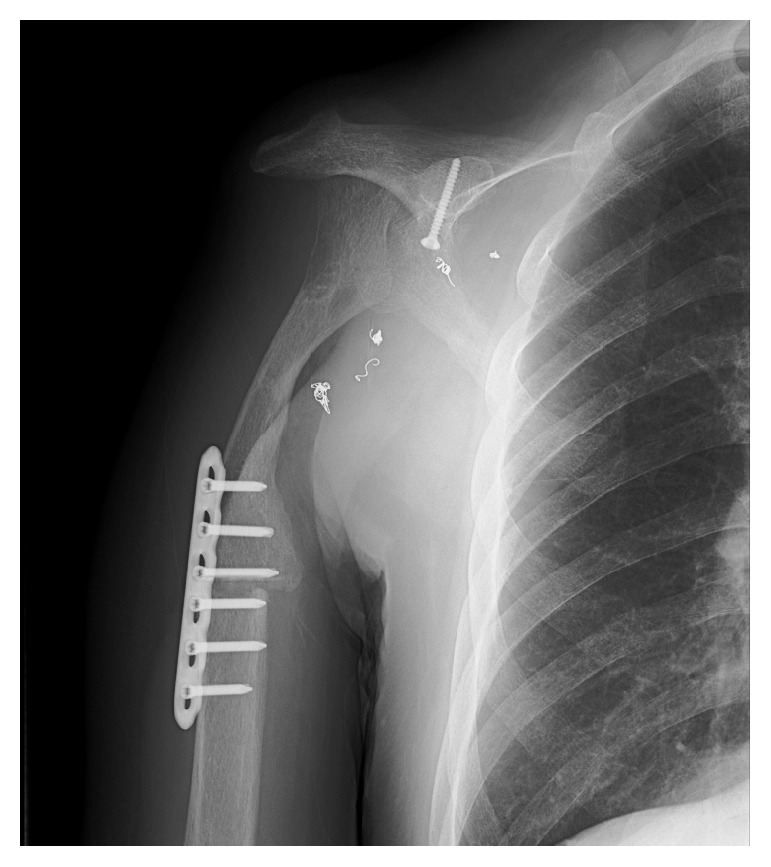
Postoperative XP. The rotated clavicle and distal humerus are fixed with a titanium plate and bony union is observed between them.

No postoperative complications were observed after the surgery. Radiographs taken 9 months after the operation showed callus formation between the clavicle and the distal humerus. The postoperative length of the reconstructed upper limb was 28 cm, 1 cm shorter than the preoperative length (Figure 3). The patient had good arm function based on the Musculoskeletal Tumor Society (MSTS) score for limb salvage evaluation with scores of 5 for pain, 3 for function, 3 for emotional acceptance, 2 for positioning of the hand, 5 for manual dexterity, and 2 for lifting ability. The total score was 20 points (67%).

**Figure 4:**
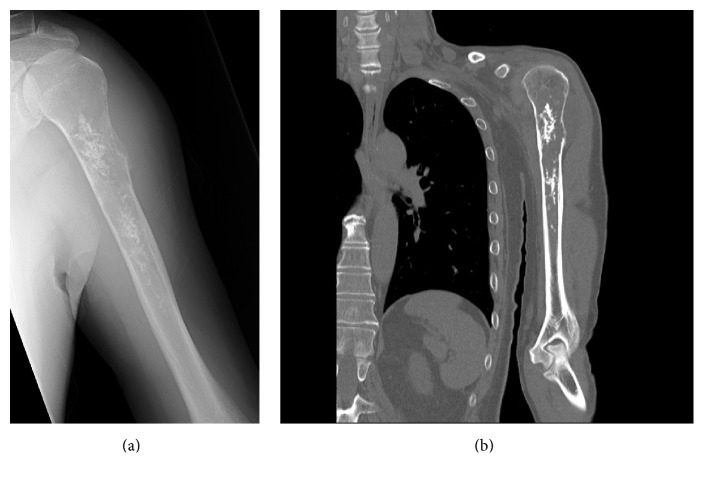
(a) A plain radiography and (b) CT of the left humerus. Bone marrow calcification and cortical expansion of the proximal humerus are observed.

**Figure 5:**
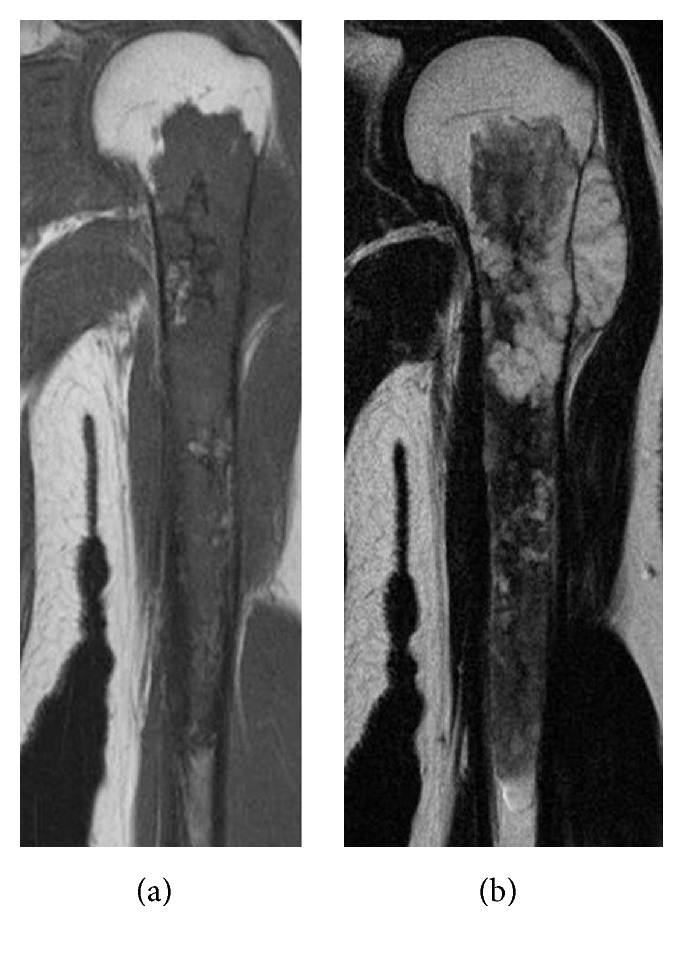
MRI findings of the left humerus. (a) T1 weighted imaging of the coronal view shows isosignals extending from the metaphysis to the diaphysis. (b) T2-weighed imaging shows intramedullary heterogenous changes with extraosseous high signals in the proximal humerus.

*Case 2*. A 66-year-old man presented to a nearby hospital, with pain in the left shoulder. He had no history of disease. The range of motion for the left shoulder was normal. Plain radiographs and CT showed intramedullary calcification of the humerus and thinning of the cortex of the proximal humerus (Figure 4). On MRI, the lesion showed low signal change on the T1 weighted image and high signal change on the T2 weighted image that extended into the soft tissue around the proximal humerus (Figure 5). Needle biopsy confirmed chondrosarcoma. We performed wide resection of the tumor, followed by a CPH reconstruction.

The length from the head to the distal mineralization was 20 cm; the right humerus was cut 23 cm below the humeral head to obtain an adequate wide margin, together with surrounded muscles of the lesion. In order to maintain the preoperative length of the humerus, we used free fibular bone grafts between the clavicle and distal humerus. No postoperative complications were observed.

**Figure 6:**
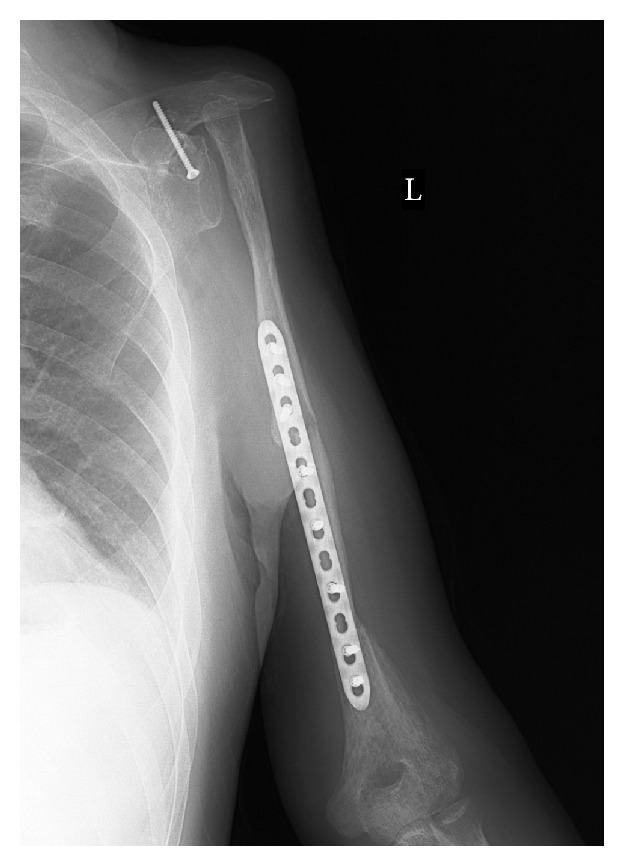
Postoperative XP. The rotated clavicle, the free fibula graft, and the distal humerus are fixed with a titanium plate, and bony union is observed among them.

Plain radiographs taken 8 months after the operation revealed bony union of the reconstructed upper limb. Its length was 32.5 cm, 0.5 cm shorter than the preoperative length (Figure 6). The patient was in good condition based on the MSTS scores for limb salvage evaluation, with scores of 4 for pain, 3 for function, 2 for emotional acceptance, 3 for positioning of the hand, 5 for manual dexterity, and 4 for lifting ability. The total score was 21 points (70%).

## 3. Discussion

This study describes two cases of CPH reconstruction that were performed in elderly individuals after wide resection of proximal humeral tumors. The MSTS functional scores after surgery indicated that CPH reconstruction was comparable to other reconstructive techniques for these patients.

The proximal humerus is a common site where bone tumors occur frequently and limb salvage surgery is the preferred surgical option whenever possible [10]. CPH reconstruction was previously performed primarily for the patients with upper extremity phocomelia [3]. Thereafter, Winkelmann began to apply the procedure for malignant tumors of the proximal humerus, as a biologic reconstruction [11]. CPH reconstruction is a type of local bone flap. The clavicle is detached medially and rotated downwards, keeping its feeding vessels intact. Thereafter, the rotated clavicle and osteotomized humerus are connected using plates and screws.

The most important aspect of this method is that the acromioclavicular joint is preserved to maintain continuation of the scapula and the clavicle. This feature contributed for postoperative shoulder stability after treatment without arthrodesis.

Several procedures exist for the reconstruction of the proximal humerus after wide resection of malignant tumors. Shoulder arthrodesis is a suitable choice for limb salvage reconstruction that uses vascularized fibular grafts. This procedure provides a stable shoulder girdle and enhances function [9]. Recently, a double-barrel vascularized fibular graft was used to decrease complications caused by the low stability of a single fibular autograft [12, 13]. However, Tsukushi et al. described problems with arthrodesis, stating that it was difficult to attach the reconstructed graft to the scapula and that prolonged postoperative immobilization was necessary to ensure bony union. They also described difficulties associated with performing thoracotomies, which are used to treat ipsilateral lung metastases, among individuals who underwent arthrodesis [9].

Replacement of the proximal humerus with a prosthesis may provide good results for patients when the deltoid muscle or rotator cuff is preserved. The use of prostheses was initially reported around 1970 [14–16]. Cannon et al. reported that proximal humeral endoprosthesis was a durable reconstruction method as it provided a stable platform for elbow and hand function [1]. In addition, it is generally the least time-consuming option for the administration of postoperative chemotherapy and radiotherapy [5]. However, infection is the most common and severe surgical complication associated with the procedure, occurring at a rate of greater than 10%. Superior subluxation of the implant has also been reported as a complication [4, 17].

Sling procedures that implement fibular grafts have been reported since early times [18]. A recent study described a procedure in which a free vascularized fibula was grafted to the proximal humerus after wide resection of a tumor, as nonvascularized grafts longer than 12 cm are more likely to cause stress fractures [19]. The tendons of the biceps femoris and the palmaris longus together were used to suspend the head of the fibula from the acromion. Although revascularization of the fibula requires skill in microscopy, the sling procedure is meritorious as it is easier to perform than an arthrodesis and there is no need for postoperative immobilization. However, some complications were reported; bony protrusion of the acromion sometimes caused pain and irritation of the skin [5] and flail shoulder without the continuity of bone triggered postoperative instability and loss of lifting ability [20].

Also, donor site morbidity after harvesting fibular graft, such as peroneal nerve palsy, toe clawing, ankle instability, and flexor halluces longus weakness has been reported [12]. To avoid this morbidity, using allograft could be considered. Some authors have reported the experience of reconstruction with allograft [6, 7], but it is not widespread in our country now.

Upon considering the aforementioned information and postoperative shoulder stability, we performed CPH reconstruction on our cases. We did not elect to perform shoulder arthrodesis since our patients had the possibilities of undergoing pulmonary metastasectomy in the future and since their physical activity was not expected to increase as they were elderly people over the age of 65. Reconstruction with a prosthesis was not appropriate because complication rates were relatively high and the procedure would not help them maintain shoulder function after resection of the deltoid muscle and rotator cuff [9, 17]. In addition, we did not adopt sling procedure to avoid donor site trouble or complex microsurgery.

Although studies of CPH reconstruction have been performed, they involved young patients, presumably because of the development of this method for limb deficiencies. Prior to this study, only one case of CPH reconstruction involving an elderly patient had been reported [8], but postoperative upper limb function was not discussed.

We did not observe postoperative complications in our patients. In previous reports, prominence of the acromioclavicular joint with skin perforations, recurrent fractures, infections, or ruptured acromioclavicular ligaments were observed after CPH reconstruction [2, 8, 21]. However, the postoperative complication rate was considered modest compared to that of other methods (Table 1).

In this study, the average postoperative upper limb function was 68.5%, which was evaluated using the MSTS scoring system. This was comparable to that of previous reports of CPH reconstruction that examined relatively younger subjects [2, 8, 9]. The upper limb function after CPH reconstruction was comparable to that of other reconstruction methods (Table 1). We particularly found CPH reconstruction to be appropriate after the excision of deltoid muscle.

In this study, the average period of bony union of the rotated clavicle to the humerus after CPH reconstruction was 8.5 months, which seemed approximately the same as previously described [9]. The literature looking into the factors affecting duration of bone union after CPH reconstruction is scarce. On the other hand, there was a report that bone union after vascularized fibular grafts was not markedly influenced by chemotherapy of radiotherapy [22]. Considering that CPH reconstruction was similar to vascularized fibular grafts at a point to preserve the blood supply of autologous bone graft, we thought that these additional therapies would not have much effect on bone union after CPH reconstruction although further studies would be needed.

In this study, the upper limb length after reconstruction was almost the same as that of the unaffected side. It is believed that shortening of reconstructed proximal humerus is beneficial for elbow and wrist function [2], but reconstruction without length discrepancy did not have any problem. Considering the cosmetic problem, reconstructions that enable the length of the upper limb to remain similar to that of its preoperative length are desirable.

In conclusion, we examined two cases of CPH reconstruction in elderly patients and the results were satisfactory. The clavicula pro humero method of reconstruction was useful not only for young patients but also for elderly patients for reconstruction after proximal humeral tumor resection.

## Figures and Tables

**Table 1:** Complication rate and MSTS score after reconstruction of proximal humerus.

Authors	Reconstruction method	Number of patients	Complication rate	MSTS score (%)
Cannon et al. [1]	Prosthesis	83	27%	63
Wafa et al. [4]	Prosthesis	34	38%	83
Sulamaa [3]	Arthrodesis	5	40%	58.3–85.0
Wada et al. [5]	Vascularized fibula graft	8	75%	79
Getty and Peabody [6]	Allograft	16	69%	70
Potter et al. [7]	Allograft	17	65%	71
	Prosthesis	16	44%	69
Kitagawa et al. [8]	Arthrodesis	4	25%	(87/1 case)
	Prosthesis	10	30%	(68/5 cases)
	Clavicula pro humero	7	14%	(71.5/2 cases)
Nishida et al. [2]	Clavicula pro humero	2	50%	80
Tsukushi et al. [9]	Clavicula pro humero	7	14%	69
*Current authors*	*Clavicula pro humero*	*2*	*0%*	*68.5*
